# An Insulin Bolus Advisor for Type 1 Diabetes Using Deep Reinforcement Learning

**DOI:** 10.3390/s20185058

**Published:** 2020-09-06

**Authors:** Taiyu Zhu, Kezhi Li, Lei Kuang, Pau Herrero, Pantelis Georgiou

**Affiliations:** 1 Centre for Bio-Inspired Technology, Department of Electrical and Electronic Engineering, Imperial College London, London SW7 2AZ, UK; taiyu.zhu17@imperial.ac.uk (T.Z.); lei.kuang18@imperial.ac.uk (L.K.); pherrero@imperial.ac.uk (P.H.); pantelis@imperial.ac.uk (P.G.); 2 Institute of Health Informatics, University College London, London NW1 2DA, UK

**Keywords:** type 1 diabetes, deep learning, insulin bolus, reinforcement learning, artificial pancreas, artificial intelligence, deep neural networks

## Abstract

(1) Background: People living with type 1 diabetes (T1D) require self-management to maintain blood glucose (BG) levels in a therapeutic range through the delivery of exogenous insulin. However, due to the various variability, uncertainty and complex glucose dynamics, optimizing the doses of insulin delivery to minimize the risk of hyperglycemia and hypoglycemia is still an open problem. (2) Methods: In this work, we propose a novel insulin bolus advisor which uses deep reinforcement learning (DRL) and continuous glucose monitoring to optimize insulin dosing at mealtime. In particular, an actor-critic model based on deep deterministic policy gradient is designed to compute mealtime insulin doses. The proposed system architecture uses a two-step learning framework, in which a population model is first obtained and then personalized by subject-specific data. Prioritized memory replay is adopted to accelerate the training process in clinical practice. To validate the algorithm, we employ a customized version of the FDA-accepted UVA/Padova T1D simulator to perform in silico trials on 10 adult subjects and 10 adolescent subjects. (3) Results: Compared to a standard bolus calculator as the baseline, the DRL insulin bolus advisor significantly improved the average percentage time in target range (70–180 mg/dL) from 74.1%±8.4% to 80.9%±6.9% (p<0.01) and 54.9%±12.4% to 61.6%±14.1% (p<0.01) in the the adult and adolescent cohorts, respectively, while reducing hypoglycemia. (4) Conclusions: The proposed algorithm has the potential to improve mealtime bolus insulin delivery in people with T1D and is a feasible candidate for future clinical validation.

## 1. Introduction

Diabetes is a group of metabolic disorders primarily characterized by elevated blood glucose (BG) levels, resulting from the dysfunction of insulin secretion. The majority of diabetes has been classified as type 1 diabetes (T1D) and type 2 diabetes (T2D) [1]. Due to the destruction of pancreatic β-cells, people living with T1D suffer from the absolute deficiency of endogenous insulin production and require long-term self BG monitoring and exogenous insulin administration.

To mimic the efficacy of natural insulin, there are two typical insulin replacements to reduce the abnormal increase of BG levels. One is slow-acting basal insulin, also known as background insulin, constantly delivered to maintain BG levels during the periods of fasting conditions. The other is fast-acting bolus insulin that aims at compensating the BG increase after meal ingestion. In general, standard basal-bolus therapy is delivered through multiple daily injections (MDIs) or continuous subcutaneous insulin infusion (CSII). An MDI regimen is usually more cost-effective than CSII, whereas the CSII treatment had been shown to effectively improves the performance of glycemic control [2]. An optimal insulin intervention for glycemic control is the key to reduce the risk of hyperglycemia and hypoglycemia and avoid short and long-term complications [3]. The settings of basal insulin, such as the pump infusion rate, can be adjusted according to historical records, while the dose of fast-acting bolus insulin is usually determined by a bolus advisor. In most cases, a bolus advisor employs an algorithm to calculate the insulin dose based on the estimation of carbohydrate in a meal and the settings of physiological parameters (i.e., insulin to carbohydrate ratio and insulin correction factor). However, due to the large variability and uncertainty in BG control, there is still a need for improvement of the algorithms and systems employed for insulin delivery [4].

Empowered by the latest advances in continuous glucose monitoring (CGM), the development of closed-loop insulin delivery systems, i.e., artificial pancreas (AP) systems, has been accelerated [5]. In an AP system, the CGM sensor measures real-time glucose concentration at a fixed frequency (e.g., every 5 min) and transmits the readings to a controller which computes the insulin doses to be delivered by a pump. AP systems have been proved to improve glycemic control in clinical trial setting and clinical practice [5]. However, its efficacy is still sub-optimal for postprandial glucose control due to the slow pharmacokinetics of existing insulin. This limitation has been compensated by the utilization of hybrid AP systems, which require meal announcement and/or meal size estimation in order to deliver an insulin bolus at meal time. Different research groups have developed advanced bolus advisors to further enhance the accuracy of insulin doses recommendations. Assuming the bolus insulin therapy in people with T1D is repetitive by nature, the use of run-to-run (R2R) control with capillary blood measurements have been proposed [6] and clinically evaluated [7]. By enhancing R2R control with CGM measurements and the artificial intelligence (AI) technique of case-based reasoning (CBR), Herrero and colleagues proposed an advanced bolus advisor which was integrated in a smartphone [8] and clinically evaluated in a free-living setting over 6 weeks [9]. In recent years, taking advantage of the increasing data availability thanks to the use of wearables and electronic health records, AI technologies are playing an important role to support decision-making systems in diabetes management [10]. Tyler et al. proposed the use of k-nearest neighbours (KNN) decision support system to provide weekly insulin dosage recommendations to adults with T1D using MDI therapy [11]. The use of KNN classifier was compared with rule-based reasoning for meal, insulin, and exercise recommendation in [12]. In [13], Aiello and colleagues proposed the use of a KNN classification algorithm to predict postprandial glucose profile due to the nominal therapy and to suggest a correction to time and/or amount of the meal bolus. Neural network have been employed to determine the insulin bolus dose [14], and a reinforcement learning (RL) method was employed to personalize the insulin treatment [15]. Combined with a RL control algorithm, the GoCARB system was presented within a AP framework to estimate carbohydrate of meals to improve glycemic control [16].

RL, a sub-field of machine learning, employs a goal-oriented agent to learn the strategies for sequential decision-making, which has been increasingly applied to glycemic control [17,18,19], and in particular, for basal insulin modulation. Deep reinforcement learning (DRL), as a recent breakthrough in machine learning, combines RL with deep learning techniques, achieving the state of art in various high-dimensional and complex tasks, such as the board-game of Go [20], autonomous driving [21], and medication dosing [22]. Deep learning techniques have been widely used to forecast future BG levels, capturing features in CGM sequences [23,24,25,26]. The major limitation of DRL applications in healthcare is that the agent explores by a trial and error process at the start of learning, which is not practical in an actual clinical settings. Fortunately, virtual environments, such as the FDA-accepted UVA/Padova T1D simulator [27], are available to perform pre-clinical trials, and which provide an ideal environment to train the RL agent. This simulator consists of a set of mathematical models, including glucose-insulin regulation, meal absorption, subcutaneous insulin absorption, continuous glucose sensor and insulin pump. It also provides a set of virtual T1D subjects, which have been proved to match clinical observations [28]. Most of the pioneering studies on advanced bolus insulin advisors have used the UVA/Padova T1D simulator to perform in silico tests [11,13,14,15,19,29]. A generalized DRL model can be trained in the simulator and then fine-tuned with subject-specific data in a clinical setting. Unlike the supervised learning model in [30], where the historical clinical data was used to fine-tune the model after in silico pre-training, an RL model requires an interactive environment to collect the latest transitions to update policy. Meanwhile, evaluating the proposed RL model without obtaining specific postprandial glucose levels is challenging. Therefore, offline analysis for the proposed RL model on in vivo data is not included in this work.

## 2. Methods

In this section, we first formulate the problem of meal insulin dosing in terms of Markov decision process (MDP) and introduce the learning algorithms to train the DRL models. Then the system architecture to implement DRL bolus advisor in an actual clinical setting is illustrated. Finally, the in silico experiments and performance metrics are presented.

### 2.1. Problem Formulation

We consider a standard DRL setup to formulate the problem of insulin bolus advisor, which employs an agent to deliver insulin and interact with the environment of diabetes $E_{D}$ in discrete timesteps. At timestep *t*, i.e., meal time, the agent first receives an observation $s_{t}$ from the sensing devices in the glucose control system and takes an action $a_{t}$ to deliver a certain amount of insulin bolus. Then the physiological state of the T1D subject transits to $s_{t+1}$ and returns the reward $r_{t}$ based on the evaluation of postprandial glucose excursion. In this regard, this problem can be modeled as a MDP with a tuple 〈S,A,P,R〉, where S is the state space, A is action space, *P* is the transition functions between states and *R* is a reward function. The policy of the agent maps the distribution of actions for a given state, which is defined by π:S→P(A). Under the policy π, the action-value function $Q^{\pi }\left(s_{t},a_{t}\right)$ is the sum of the discounted future reward with the current state $s_{t}$ and chosen action $a_{t}$. The target of RL is to obtain the optimal policy to gain maximum reward. Moreover, a policy μ can be modeled by mapping the state to a specific action in a deterministic environment as in our case: μ:S→A. In this regard, the estimated target policy can be solved by the Bellman equation:

(1) $$Q^{\mu }\left(s_{t},a_{t}\right)=E_{r_{t},s_{t+1}∼E_{D}}\left[R\left(s_{t},a_{t}\right)+\gamma Q^{\mu }\left(s_{t+1},\mu \left(s_{t+1}\right)\right)\right],$$

where γ is a discount factor within the range of [0,1], and $Q^{\mu }$ can be learned by off-policy. In the context of glycemic control, the input and output of the algorithm are the current observation of physiological states and insulin bolus suggestion, respectively. The objective is to obtain the meal insulin bolus that optimizes glycemic control by solving Equation (1). Safety constraints on insulin suggestions are required to avoid undesirable glucose events in a clinical setting.

In this problem, the insulin bolus varies largely depending on the meal ingestion and will significantly influence the postprandial BG levels. It is difficult to discretize the range of feasible bolus insulin doses as the action set with value-based DRL, such as deep Q-networks [31]. Too many intervals will exponentially slow the training process, while a small action set can degrade the performance due to the variability of meals. Alternatively, we could get an estimation of the dosage with a bolus calculator and then vary a continuous range (e.g., ±30%) around this value. Therefore, we introduce an actor-critic method to enable a continuous action-space for the agent-based on deterministic policy gradient [32]. The critic function Q(s,a) is recursively learned by the Bellman equation in Equation (1). With the initial distribution *J* of the parameters, the actor function μ(s) is updated as:

(2) $$\nabla_{\theta^{\mu }}J\approx E_{s_{t}∼\rho }[\nabla_{a}Q\left(s,a|\theta^{Q}\right){|}_{s=s_{t},a=\mu \left(s_{t}\right)}\nabla_{\theta^{\mu }}\mu \left(s|\theta^{\mu }\right){|}_{s=s_{t}}],$$

where $\theta^{Q}$ and $\theta^{\mu }$ are the parameters of critic and actor, respectively, ρ is the state-visitation distribution. Specifically, the actor decides how many units of bolus insulin to deliver for the current physiological state, whilst the critic determines how good the action was taken and tells the actor how to adjust.

#### 2.1.1. Deep Neural Networks

To learn a generalized policy in a large action-state space, we use deep neural networks (DNNs) as the non-linear approximators to parameterize the actor and critic following the method of deep deterministic policy gradient (DDPG) [33], which means $\theta^{Q}$ and $\theta^{\mu }$ in Equation (2) become the weights of neural networks. Following the success of the DRL on human-level control [31], we employ replay memory M and fixed target networks to further improve the stability of the proposed methods. M stores last *N* transitions with a tuple $\langle s_{t},a_{t},r_{t},s_{t+1}\rangle$ to provide experience samples for off-policy learning. Using fixed target networks, we have separated neural networks to calculate targets during the model training. Thus, the target neural networks of actor $Q^{\prime }$ and critic $\mu^{\prime }$ are obtained by copying weights of current actor *Q* and critic μ with a fixed period T, using soft update [33]. Referring to Bellman Equation (1) and transition tuples, the loss of critic is formulated as follows:

(3) $$L\left(\theta^{Q}\right)=E_{s_{t}∼\rho }[\left(r_{t}+\gamma Q^{\prime }\left(s_{t+1},u^{\prime }\left(s_{t+1}|\theta^{u^{\prime }}\right)|\theta^{Q^{\prime }}\right)-Q{\left(s_{t},a_{t}|\theta^{Q}\right)}^{2}\right].$$

**Figure 1:**
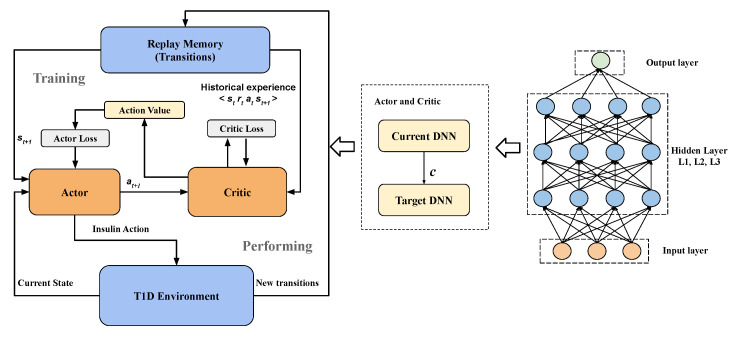
The block diagram of the proposed DDPG model with the actor-critic architecture.

Particularly, the value-based algorithm includes calculating the temporal-difference (TD) error to update the weights of critic neural network with Equation (3), while the policy-based part is using the outcomes of critic and Equation (2) to update actor neural network. Repeatedly updating the weights of critic and actor DNNs, the model can learn the policy to maximize the expected reward by delivering optimal bolus, as depicted in Figure 1. We instantiate DNNs with three fully connected hidden layers for both the actor and critic. The details of DRL elements with respect to glucose control are described in the following sections.

#### 2.1.2. Agent States And Actions

For the standard bolus calculator in AP systems with CGM and insulin pumps [34], a relatively empirical formula is used to calculate the insulin dose:

(4) $$Bolus_{t}^{*}=\frac{CHO_{t}}{ICR}+\frac{G_{t}-G^{T}}{ISF}-IOB_{t},$$

where $CHO_{t}$ is the total carbohydrate amount of the meal ingestion (gram), ICR is the insulin-to-carbohydrate ratio (g/IU); ISF is the insulin sensitivity factor (mg/L/IU); $G_{t}$ is the current reading of BG level from CGM (mg/dL); $G^{T}$ is the target BG level; and IOB is insulin on board. The ISF is commonly multiplied by a portion to adapt correction insulin [35]. IOB can be estimated by the previous bolus dose using various methods, e.g., dynamic rule-based algorithms [36], artificial neural networks [37], circadian insulin sensitivity variation [38], or a simple formula as:

(5) $$IOB_{t}=Bolus_{t-1}*\max(0,\left(1-\frac{\left(ts_{t}-ts_{t-1}\right)}{T_{IOB}}\right),$$

where ts is the time of bolus delivery, and $T_{IOB}$ is a manually defined interval to indicate the active time. Note that ts stands for the time sampled by the CGM at fixed intervals, e.g., every 5 min. In real clinical practice, these parameters $\left\{ICR,ISF,T_{IOB}\right\}$ are not time-varying and might depend on the physiological state of the subject. This underline non-linear function can be approximated by the actor function with the weights of DNNs $\mu (\theta^{\mu })$. Furthermore, considering DNNs have the superior capability of representation learning [39], we extend the $G_{t}$ into a series of historical record of CGM $G_{t}$ to extract more features from input states, such as the BG trends. As a result, the agent state at timestep *t* is denoted as:

(6) $$s_{t}=\left\{G_{t},CHO_{t},ts_{t},IOB_{t}\right\}\in S,$$

where $G_{t}\in R^{1\times L}$ contains a number (*L*) of BG measurements from CGM.

In most cases, the action space of DDPG methods is defined by a range. The range of insulin bolus could be relatively large due to the uncertainty of meal carbohydrate intake. To improve the convergence of the training, we define the bolus actions from DDPG as:

(7) $$Bolus_{t}=\left[\frac{CHO_{t}}{ICR},\frac{G_{t}-G^{T}}{ISF},-IOB_{t}\right]*\mu {\left(s_{t}|\theta^{\mu }\right)}^{T}\in A$$

where the output of the actor function $\mu \left(s_{t}|\theta^{\mu }\right)=\left[g_{ICR},g_{ISF},g_{IOB}\right]$ is defined as a three-element vector, consisting of the gains to respectively adapt ICR, ISF, and IOB for the standard bolus calculator. In this case, the range of the gains is defined as [0.2, 2], to reduce or amplify the bolus insulin. The action is the same as the stand bolus calculator when $\mu \left(s_{t}|\theta^{\mu }\right)=\left[1,1,1\right]$.

#### 2.1.3. Reward Function

The goal of a bolus advisor is to maximize postprandial BG levels in the target zone, i.e., [70, 180] mg/dL while minimizing the the occurrence of hypoglycemia [29,40] To guide the agent to achieve this goal, the positive rewards are applied to the time in range (TIR) zone. Employing CGM systems, we obtain a series of postprandial BG levels with a fixed sampling period, which allows us to assign a reward value for each postprandial BG reading then sum them up:

(8) $$r_{t}=\frac{1}{ts*-ts_{t}}\sum_{k=ts_{t}}^{ts*}f_{R}\left(G_{k}\right),$$

where $ts*=\min(ts_{t+5h},ts_{t+1})$. If the time interval between two successive states (meal ingestion) is too large, we only consider 5-h postprandial period after the current meal [29]. The discrete reward is formulated as:

(9) $$f_{R}\left(G_{k}\right)=\left\{\begin{array}{c} 0.5,\;\;\;\;\;\;70\leq G_{k}\leq 180,\\ -0.8,\;\;180<G_{k}\leq 300,\\ -1,\;\;\;\;\;\;300<G_{k}\leq 350,\\ -1.5,\;\;30\leq G_{k}<70\\ -2,\;\;\;\;\;\;\mathrm{else}. \end{array}\right.$$

**Figure 2:**
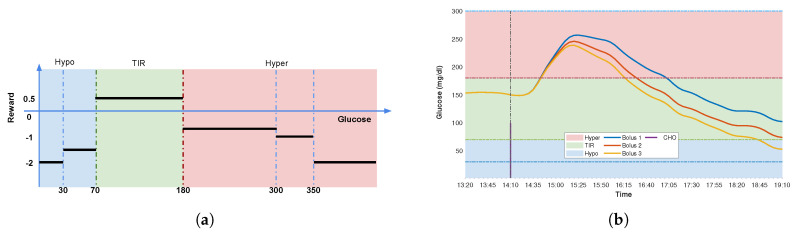
Illustration of proposed reward function to determine the performance of the action that was taken. (**a**) Step function to calculate the reward for the discrete BG values after dietary intake, referring to Equation (9). The blue, green, and red regions stand for hypoglycemia, normoglycemia, and hyperglycemia zones, respectively; (**b**) Postprandial glucose curves corresponding to three different bolus and same variability.

Figure 2a depicts the proposed reward function. Figure 2b shows a comparison of the postprandial excursions corresponding to three different insulin bolus $\left\{Bolus_{1},Bolus_{2},Bolus_{3}\right\}$ for the same meal and variability. The TIR and rewards for the three insulin boluses are {53.3%, 65.0%, 58.3%} and {−0.107, 0.045, −0.112}, respectively. Although $Bolus_{3}$ obtains better TIR than $Bolus_{1}$, its reward is smaller than that of $Bolus_{1}$, which is due to the increase of hypoglycemia. People with hypoglycemia episodes are at major risk of acute short-term complications (e.g., coma), which is in general less preferable than hyperglycemia [41].

### 2.2. Two-Step Learning Framework

Collecting large sets of clinical data is often expensive, and evaluating algorithms on human subjects without pre-clinical validation, or proper safety constraints, might be dangerous. To this end, we propose a two-step learning framework, employing the UVA/Padova T1D simulator and recent advances in DRL. In particular, at the first step, the agent is allowed to explore random actions by adding Gaussian noise N(0,0.3) and constantly interact with the simulator. For this purpose, we use the average T1D subject provided by the simulator. To obtain a population DRL model, the agent performs long-term off-policy learning until the loss of critic converges. In the second step, the weights of the population model are used to initialize a personalized model for each individual by transfer learning. Then the models are further fined-tuned by using subject-specific data with safety constraints in a short-term training period of $T_{p}$. Here we use a simple constraint during in silico validation: the action gain is limited to be greater than 1 if current BG enters hyperglycemia and less than 1 for hypoglycemia. In a clinic practice, more advanced constraints could be used, such as a interval arithmetic-based dynamic insulin constraint proposed by Liu et al. [42]. During the fine-tuning, the data is collected with a form of transitions, i.e., a tuple of $\langle s_{t},a_{t},r_{t},s_{t+1}\rangle$. According to Equation (6), such a transition requires multiple data fields, including CGM measurements, estimated carbohydrate of meal ingestion, mealtime, and dosages of insulin bolus. These data fields are available with the proposed system architecture in Section 2.3, where carbohydrate estimation is manually entered, while other data can be collected automatically. Finally, we test the personalized models on separate testing sets.

To accelerate the training process, we adopt a variant of prioritized memory replay to sample mini-batches of the transitions [43]. The priority, i.e., the probability of sampling a transition Pr(i), is based on the magnitude of the TD error $\delta_{i}$, which is denoted as:

(10) $$Pr\left(i\right)=\frac{\left(\right|\delta_{i}{\left|+\epsilon \right)}^{\alpha }}{\sum_{n=1}^{N}\left(\right|\delta_{n}{\left|+\epsilon \right)}^{\alpha }},$$

where *N* is the total number of transitions in replay memory; ϵ is a small positive constant to guarantee that the transitions with zero TD error can also be sampled; and α is the degree of using prioritization (α=0 stands for uniform sampling). To remove the bias of the prioritized sampling and improve convergence, a set of importance weights is introduced and normalized as: $w_{i}={\left(N*Pr\left(i\right)\right)}^{-\beta }/\max_{n}w_{n}$, where β is the degree to compensate the prioritization (β=1 means full compensation). The details of the complete training algorithm are presented in Algorithm 1. The hyper-parameters are listed in Table A1.
**Algorithm 1** DDPG Insulin Bolus Advisor.1:**Input:** average environment $E_{a}$, individual environment $E_{i}$, safety constraints C, update period T.2:**if** personalized training **then**3:    Initialize the weights $\theta^{Q}$, $\theta^{\mu }$ from the population model, N=0, $E_{D}=E_{i}$,4:**else**5:    Randomly initialize the weights $\theta^{Q}$, $\theta^{\mu }$ for actor *Q* and critic μ, C=ϕ, $E_{D}=E_{a}$6:**end if**7:Copy the weights to the target networks $Q^{\prime }$ and $\mu^{\prime }$: $\theta^{Q^{\prime }}\leftarrow \theta^{Q}$, $\theta^{\mu^{\prime }}\leftarrow \theta^{\mu }$8:Initialize empty replay memory M with the volume of *N* and prioritization9:**repeat**10:    Observe state $s_{t}$ from *E*, select action by actor $a_{t}=\mu \left(s_{t}|\theta^{\mu }\right)+N$,11:    **if** $a_{t}$ subject to C**then** execute $a_{t}$ in *E***else** restrict $a_{t}$ by C **end if**12:    Observe state $s_{t+1}$, calculate reward $r_{t}$, store the transition $\left(s_{t},a_{t},r_{t},s_{t+1}\right)$ in M13:    Sample a mini-batch from M by priority Pr14:    Calculate the loss of critic $L(\theta^{Q})$ and update the weights $\theta^{Q}$ with importance weights *w*15:    Calculate TD error, update Pr and *w*16:    Perform a gradient descent $\nabla_{\theta^{\mu }}J$ to update $\theta^{u}$17:    **if** *t* mod T=0**then** soft update: $\theta^{Q^{\prime }}\leftarrow \tau \theta^{Q}+\left(1-\tau \right)\theta^{Q^{\prime }}$, $\theta^{\mu^{\prime }}\leftarrow \theta^{\mu }+\left(1-\tau \right)\theta^{\mu^{\prime }}$ **end if**18:**until** the loss of critic converges or t = $T_{p}$

### 2.3. System Architecture

**Figure 3:**
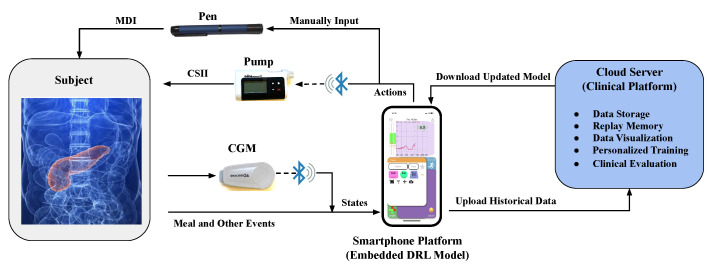
System architecture to evaluate the DRL models in an ambulatory clinical setting.

The use of smartphone applications in diabetes management has seen promising results [44]. Several projects aiming at enhanced glucose management have used CGM and smartphone applications, such as DiAs [45], PEPPER [42], ABC4D [8], and iAPS [46]. The proposed algorithm will also be evaluated as the insulin bolus advisor on a smartphone platform for T1D self-management during an upcoming clinical trial. The system architecture including the DRL algorithms is presented in Figure 3.

In this system architecture, the central component is a smartphone application that collects real-time data from two sources. One is the wearable sensors for acquiring physiological and physical data via Bluetooth, and the other is a manual log for recording any exogenous events, such as meals, exercise, and health conditions [47]. The multi-modal data in Equation (6) comprises the CGM readings, carbohydrate content, and meal ingestion time, all of which can be accessed by the algorithm inside the application. The DRL models in this work are developed by TensorFlow [48] and converted to the modules of mobile devices by TensorFlow Lite [24,49]. Based on the input state, the embedded DRL advisor can calculate a corresponding action, i.e., the gain of meal insulin bolus, to assist users to control the insulin pump with CSII, or insulin pen with MDI. The historical data is automatically uploaded to a cloud server for monitoring and backup purposes. To monitor the BG levels and strategies of the insulin bolus advisor, we build a visualization platform for clinicians on the cloud server. Moreover, the uploaded data forms a pool of the replay memory, where the DRL models can be further updated using new data and personalized training. Finally, the application automatically fetches the updated model and saves it in local storage.

### 2.4. In Silico Validation

To validate the performance of the proposed algorithm, we use a customized version of the UVA/Padova T1D simulator [27] as the platform to conduct pre-clinical trials. For this purpose, we tested the models on 10 adult and 10 adolescent virtual subjects. Additional intra-day variability was introduced to better emulate real-life conditions [50]. In particular, we employ a daily pattern with three realistic meals: breakfast, lunch, and dinner. The time and carbohydrate content are as follows: 7 a.m. (70 g), 2 p.m. (110 g), and 9 p.m. (90 g), and the duration of each meal is set to 15 min. The variability of meal-time and meal-size are set to StD=30 min and CV=10%, respectively. Besides, we consider that the subjects are likely to under or over-estimate the carbohydrate content of meals by 70% and 110%, respectively. The intra-subject insulin sensitivity variability is set to 30% following a sinusoidal function [50]. Although a single dose of basal insulin might not be optimal to cover the basal insulin requirements due to the presence of intra-subject variability, this a common practice in people with type diabetes on MDI. Hence, we wanted to test the viability of our proposed approach on this subpopulation, which represents the majority of people with T1D.

In this work, we first use the average virtual subjects to train a generalized model over a long period until the performance, i.e., the learning curve, is stable. This step includes random exploration, which needs to be done in the simulator. With proper safety constraints and initialisation, personalized training can be then conducted in an actual clinical setting. Here, we perform the second training step on 180 simulated days (6 months) to fine-tune the personalized model in the simulator. This setting is determined by the convergence of the models, i.e., the learning curves. The actual computational time of model training is short, which is around 10 milliseconds for each step [24], but it takes a long time to collect transitions in the training sets, since there are only a few meal events (i.e., 3–4 transitions) per day. If waiting 6 months is considered too long to converge to an optimal performance in an actual clinical setting, it is possible to use a larger learning rate or stop training earlier. However, in this case, the model could achieve sub-optimal performance. Finally, we test the personalized model in a period of 90 days (3 months), and the new simulations are generated by the same meal protocol. To make a fair comparison between the proposed model and baseline algorithms, the same scenarios and randomness seed of variability are saved and used for each evaluated method.

### 2.5. Performance Metrics

To measure the performance of glycemic control, we use a set of commonly employed metrics in AP clinical trials [40]. The main objective of glucose management systems is to maintain the BG levels in a target range and minimising hypoglycemia. Therefore, the percentage time in range (TIR) of [70, 180] mg/dL is an intuitive metric, which indicates the time that the BG levels of a subject are within the normoglycemia zone. Correspondingly, time below range (TBR) (BG < 70 mg/dL) and time above range (TAR) (BG > 180 mg/dL) stand for time spent in hypoglycemia and hyperglycemia, respectively. The mean BG values (Mean), coefficient of variation (CV), low blood glucose index (LBGI), and high blood glucose index (HBGI) are also used to present a comprehensive evaluation. Furthermore, control-variability grid analysis (CVGA) [51] is employed to visualize the glycemic outcomes by plotting the extreme (minimum/maximum) BG values on a grid with 9 zones, which has been widely used to compare the efficacy of different algorithms for in silico and clinical trials. The points in A+B zones stand for optimal glycemic control in AP systems.

## 3. Results

In order to evaluate the performance of the proposed algorithm, we employed a baseline method consisting of the standard bolus calculator (SBC) with fixed parameters (Equation (4)) [35]. The results of personalized DRL models and the baseline method are presented as Mean±StD. In particular, we use the paired *t*-test to compute *P* values to analyze the statistical significance, and the normality of data distribution is tested using histograms. Table 1 and Table 2 show the glycemic outcomes for the adult cohort (*n* = 10) and adolescent cohort (*n* = 10), respectively, over 3 months. It is to be noted that the DRL algorithm achieves better performance than the SBC for every evaluated metric. The TIR results have been significantly enhanced for the adult and adolescent cohorts with a significant decrease in hypoglycemia and hyperglycemia. The mean BG level is also improved for the adolescent cohort and maintained for the adult cohort. Finally, the LBGI and the HBGI, as the key metrics for measuring the risk of hypoglycemia and hyperglycemia, are largely improved.

**Figure 4:**
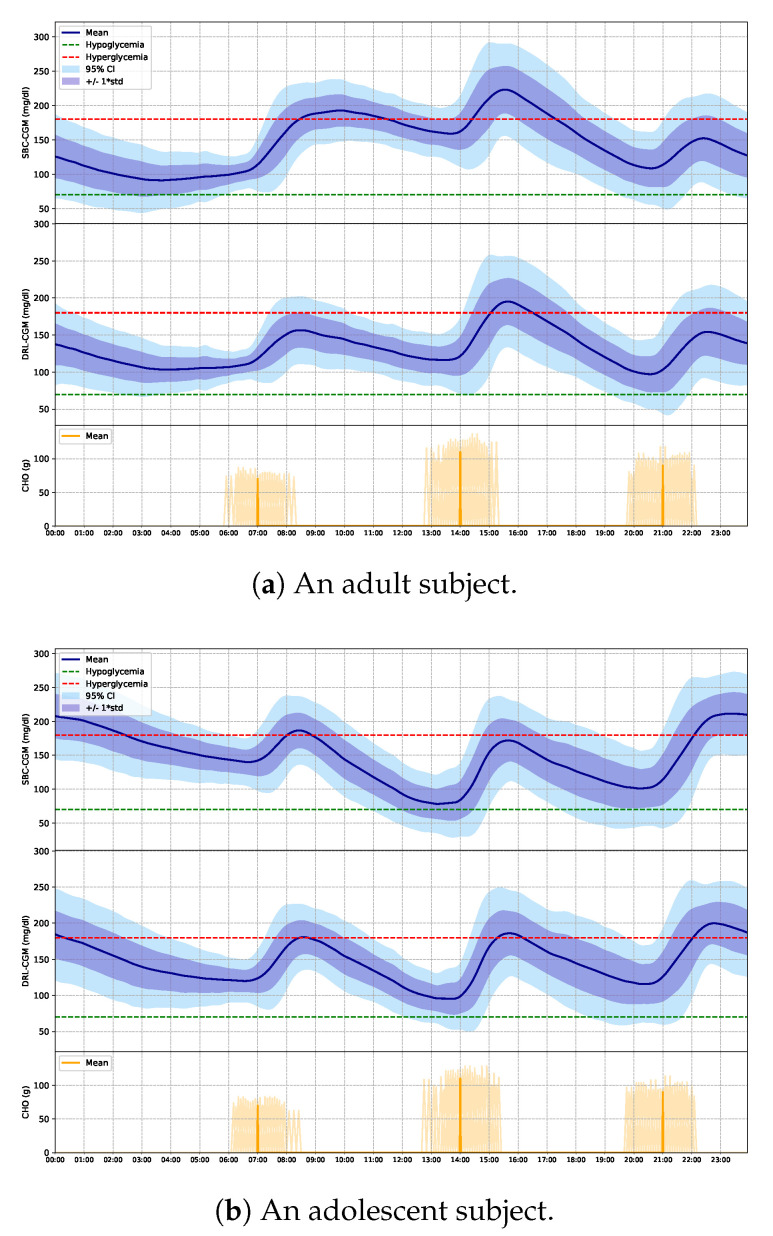
Graphical example of the improvement on glycemic control of the DRL algorithm over the SBC method. (**a**,**b**) show the performance of an adult and adolescent subject, respectively. From top to bottom, each plot shows the daily glucose trajectory of SBC and DRL and distribution corresponding to three meal ingestion over 3 months. The thresholds of hyperglycemia and hypoglycemia are displayed in red and green dashed lines. The solid blue lines indicate the average BG levels. The blue and purple shades indicate the 95% confidence interval (CI) and standard deviation, respectively.

Figure 4 shows an average glucose profile over a 24-h period for two chosen adult and adolescent subjects to illustrate the DRL algorithm improvement compared to the SBC method. After learning the personalized strategies, the well-trained DRL agent delivers an optimal insulin bolus that effectively reduced the postprandial hyperglycemia without increasing hypoglycemia.

**Figure 5:**
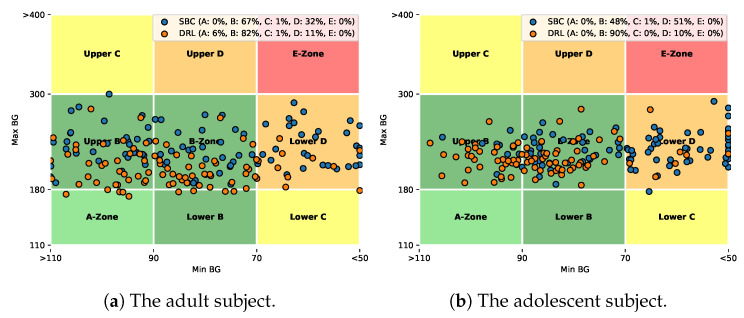
CVGA plots comparing the SBC (orange dots) and DRL (blue dots) methods corresponding to a chosen adult subject (**a**) and a chosen adolescent subject (**b**) over a three-month scenario.

Figure 5 depicts the corresponding CVGA plots for the chosen adult and adolescent subjects. Here we customized the CVGA plots, where each dot stands for the glycemic performance over 24 h, i.e., daily glucose trajectory of the same subject. The results, i.e., the distribution of the scattering dots, are consistent with the glucose profile in Figure 4. It is worth noting that, compared to the SBC method, the percentage improvement in the A+B zone increased from 67% to 88% for the adult subject and from 48% to 90% for the adolescent subject. The dot distribution of the DRL method shifted towards bottom-left corner, which is an indicator of good glycemic control.

## 4. Discussion

In this work, we proposed a novel algorithm for meal insulin bolus dosing based on deep reinforcement learning (DRL) and continuous glucose monitoring (CGM) data. The results presented in Table 1 and Table 2 show that the DRL algorithm achieves good in silico glycemic control on a customized version of the the FDA-accepted UVA/Padova T1D simulator. Compared to compared to a standard bolus calculator, the proposed methods achieves a significant improvement in TIR and hypoglycemia reduction for both, the adult and adolescent virtual cohort. Using the system architecture in Figure 3, the proposed method can be implemented on a smartphone application and updated by the cloud server without much engineering work. Therefore, it suggests that the DRL algorithm has the potential to improve meal bolus insulin bolus delivery for T1D subjects in an actual clinical setting.

To validate the robustness of the proposed method, we introduced additional intra-day variability to the in silico trials and tested the algorithm on 20 different subjects. As depicted in Figure 4a, for the chosen adult, the DRL algorithm reduced postprandial hyperglycemia after lunch, while the mean BG levels before dinner time remains above the hypoglycemia threshold thanks to the strict reward setting in the low BG zone (Figure 2). However, the improvement by optimal insulin bolus is less significant in some extreme scenarios, e.g., a highly insulin-sensitive subject ingesting a meal with high carbohydrate content. Thus, adaptive control of basal insulin and glucagon (optional) by DRL would be helpful in these cases in T1D subjects wearing insulin pumps [52], which is part of our future work. Although we introduced variability into carbohydrate misestimation, it might be worth evaluating separately the effects of under or over-estimation of carbohydrate content and investigating how these errors will influence the final strategies by the proposed DRL model.

Although the proposed algorithm outperforms the SBC baseline in each evaluated metric, there are some limitations in this study. Compared to a real clinical setting, using the simulators might overestimate the efficacy of the evaluated methods. There are many uncertainties and noises in the real world, such as the artifacts of the CGM systems, physical activities, and health conditions, which may influence BG levels and degrade the control algorithms. Consequently, future work includes the clinical validation of the proposed algorithm, while incorporating more data fields into the agent states, such as vital signs from an activity wrist band. It is worth noting that deep learning is particularly suited for extracting feature maps from time-series raw data. The major limitation is the high demand for training data, which might be difficult to obtain in a clinical setting, e.g., a 6-month training phase. Although we largely accelerate the training process by the two-step learning framework and prioritized memory replay, the training efficiency needs to be further enhanced. With the rapid development of DRL techniques in recent years, many latest advances could provide solutions to this issue, such as model-based DRL [53] and safe off-policy training [54]. Although the idea of evaluating the proposed RL algorithm on retrospective clinical data might sound very appealing, this is still an open problem in the RL research community (i.e., offline learning) and we think that it is out of the scope of this work [55]. However, we might consider it in the future if the right tools become available. Finally, at the current stage, the model training is performed on the cloud server. In future work, we consider converting the model by the framework of smartphone operating systems, such as iOS, and locally training the DRL model by real-time data and the central processing unit of the smartphone.

To the best of our knowledge, this work is the first attempt employing DRL to develop personalized insulin bolus advisor in T1D. Although many pioneering studies have used the UVA/Padova T1D simulator to develop glycemic control algorithms, the different settings in meal-protocols, variability, randomness in the scenarios make it challenging to perform a direct head-to-head comparison between the existing works. In addition, sometimes the existing algorithms are evaluated in combination with basal insulin control [15,19]. Hence, we evaluated the proposed DRL algorithm with commonly employed metrics to comprehensively assess its performance. This data-driven algorithm also has the potential to be applicable to support people with T2D on insulin. However, this requires further study and will be the subject of future work.

## 5. Conclusions

In this work, we proposed a novel meal insulin bolus advisor using DRL techniques and CGM data. The model is based on the actor-critic DDPG architecture with multiple DNNs and trained by the two-step learning framework and prioritized memory replay. When compared to the standard therapy of insulin bolus calculation, the experimental results of the in silico trials indicate promising performance of the DRL model, which significantly improved the time in the target range and reduced the risk of hypoglycemia and hyperglycemia on a virtual cohort of people with T1D. The well-trained model can be easily embedded into smartphone applications, which provides a feasible solution for future clinical trials.

## Figures and Tables

**Table 1:** Glycemic control metrics evaluating the performace of the DRL and SBC algorithms on the 10-adult virtual cohort. Statistical significance is indicated as ${}^{†}$ for p≤0.01.

Method	TIR (%)	TBR (%)	TAR (%)	Mean (mg/dL)	CV (%)	LBGI	HBGI
SBC	74.1±8.4	5.5±1.9	20.2±8.2	138.6±11.5	34.8±4.8	1.5±0.5	4.1±1.7
DRL	$80.9\pm 6.9{\;}^{†}$	$1.9\pm 1.5{\;}^{†}$	17.0±6.1	138.1±7.5	$31.1\pm 5.3{\;}^{†}$	$0.7\pm 0.4{\;}^{†}$	3.6±1.2

**Table 2:** Glycemic control metrics evaluating the performace of the DRL and SBC algorithms on the 10-adolescent virtual cohort. Statistical significance is indicated as * for p≤0.05 and ${}^{†}$ for p≤0.01.

Method	TIR (%)	TBR (%)	TAR (%)	Mean (mg/dL)	CV (%)	LBGI	HBGI
SBC	54.9±12.4	6.5±3.5	38.5±13.0	167.5±25.3	40.7±6.1	2.4±1.7	9.2±4.9
DRL	$61.6\pm 14.1{\;}^{†}$	$4.3\pm 2.4{\;}^{*}$	$34.1\pm 13.6{\;}^{*}$	161.6±24.7	$38.6\pm 7.5{\;}^{*}$	$1.3\pm 0.8{\;}^{*}$	$8.0\pm 4.8{\;}^{*}$

## Abbreviations

The following abbreviations are used in this manuscript:

AI	Artificial Intelligence
AP	Artificial Pancreas
BG	Blood Glucose
CBR	Case-based Reasoning
CGM	Continuous Glucose Monitoring
CSII	Continuous Subcutaneous Insulin Infusion
CV	Coefficient of Variation
CVGA	Control-variability Grid Analysis
DDPG	Deep Deterministic Policy Gradient
DNN	Deep Neural Network
DRL	Deep Reinforcement Learning
HBGI	High Blood Glucose Index
ICR	Insulin-to-carbohydrate Ratio
IOB	Insulin on Board
ISF	Insulin Sensitivity Factor
KNN	K-nearest Neighbours
LBGI	Low Blood Glucose Index
MDI	Multiple Daily Injection
MDP	Markov decision process
R2R	Run-to-run
RL	Reinforcement Learning
SBC	Standard Bolus Calculator
T1D	Type 1 Diabetes
T2D	Type 2 Diabetes
TAR	Time Above Range
TBR	Time Below Range
TIR	Time In Range

## Appendix A. Hyper-Parameters

Table A1 lists the hyper-parameters of the DRL model in this work. It is noted that we used the same hyper-parameters for all the simulated subjects.

**Table A1:** List of hyper-parameters.

Parameter	Value
The length of CGM measurements *L*	6
The hidden units of DNNs	[200, 200, 10]
The learning rate of the actor	0.0001
The learning rate of the critic	0.0001
The size of replay memory *N*	500
Batch size	32
Soft replacement τ	0.01
Target network update period T	100
Discount factor γ	0.9
The degree of prioritization α	0.6
Compensation factor β	0.4→1
Priority constant ϵ	0.00001
